# TRANSOLECRANON FRACTURE-DISLOCATION: CONCEPTS AND FUNCTIONAL RESULTS OF SURGICAL TREATMENT

**DOI:** 10.1590/1413-785220233101e255572

**Published:** 2023-04-17

**Authors:** José da Mota, Sebastião Alves da Cruz, Leandro Furtado De Simoni, Diego Salzer Reis Zimmermmann, Fernando Brandão Andrade-Silva, Adriano Fernando Mendes

**Affiliations:** 1 Universidade Federal de Juiz de Fora, Hospital Universitário, Serviço de Ortopedia e Traumatologia, Juiz de Fora, MG, Brazil.; 2 Hospital Maternidade Therezinha de Jesus, Serviço de Ortopedia e Traumatologia, Juiz de Fora, MG, Brazil.; 3 Universidade de São Paulo, Faculdade de Medicina, Instituto de Ortopedia e Traumatologia (IOT-FMUSP), São Paulo, SP, Brazil.

**Keywords:** Orthopedic Procedures, Elbow, Olecranon, Fractures, Bone, Joint Dislocations, Procedimentos Ortopédicos, Cotovelo, Olécrano, Fraturas Ósseas, Luxações articulares

## Abstract

**Objectives:**

This study aimed to evaluate the functional results of the treatment protocol for the treatment of transolecranon fracture-dislocation, by surgical reduction and osteosynthesis with plate and screws, in patients attended at a referral hospital for orthopedic trauma, with a minimum follow-up period of six months.

**Methods:**

Twenty-five individuals treated surgically from January 2014 to November 2018 were selected for a primary observational longitudinal study using questionnaires to assess upper limb and elbow function (DASH and MEPS), quality of life (SF-12), pain (visual analog scale - VAS), and radiographic evaluation in anteroposterior and lateral views of the elbow.

**Results:**

Fifteen patients were male, and the mean age was 46.8 years. All participants had their fractures consolidated, with no radiolgraphic signs of implant failure, or degenerative arthritis. Mean range of motion was reduced relative to the contralateral limb: 102.6º for flexion-extension and 132.8º for pronation-supination. The mean MEPS and DASH scores were 89.6 and 16.5 respectively. There was no residual pain in 84% of the cases according to the VAS.

**Conclusion:**

The surgical treatment proposed for transolecranon fracture-dislocations showed satisfactory results according to MEPS, DASH scores and quality of life measures. ***Evidence Level IV; Retrospective observational study.***

## RESUMO

**Objetivo:**

Avaliar os resultados funcionais do protocolo de tratamento da fratura-luxação transolecraniana, por redução cirúrgica e osteossíntese com placa e parafusos, nos pacientes atendidos em hospital de referência para trauma ortopédico, com seguimento mínimo de seis meses.

**Métodos:**

vinte e cinco indivíduos tratados cirurgicamente de janeiro de 2014 a novembro de 2018 foram selecionados para um estudo longitudinal observacional primário, utilizando questionários para avaliar a função do membro superior e cotovelo (DASH e MEPS), qualidade de vida (SF-12), dor (visual escala analógica - EVA), e avaliação radiográfica nas incidências anteroposterior e perfil do cotovelo.

**Resultados:**

Quinze pacientes eram do sexo masculino e a média de idade foi de 46,8 anos. Todos os participantes tiveram suas fraturas consolidadas, sem sinais radiográficos de falha do implante ou artrite degenerativa. A amplitude média do movimento foi reduzida em relação ao membro contralateral: 102,6º para flexo-extensão e 132,8º para pronossupinação. Os escores médios de MEPS e DASH foram 89,6 e 16,5, respectivamente. Não houve dor residual em 84% dos casos de acordo com a EAV.

**Conclusão:**

O tratamento cirúrgico proposto para a fratura-luxação transolecraniana apresentou resultados satisfatórios de acordo com MEPS, escores DASH e medidas de qualidade de vida. ***Nível de evidência IV; Estudo observacional retrospectivo.***

## INTRODUCTION

Elbow fracture-dislocations (EFD), although relatively common occurrences in elbow trauma (between 10 and 20%),^1^ are considered complex and unstable injuries for treatment, especially due to the osseus and soft lesions. The objective of its treatment is to achieve a stable, painless joint with a functional range of motion.^2-4^

However, the functional results of this treatment varies according to the subtype of EFD.^3,5,6^

Transolecranon fracture-dislocations (TFD) are a subgroup of EFD. They were initially described by Biga and Thomini^7^ as a complex injury associated with high-energy trauma.^7^ For Ring^8^, in TFD there is anterior translation of the forearm in relation to the distal humerus, without dissociation of the proximal radioulnar, rarely injuring the radial head or annular ligament, which distinguishes it from an anterior Monteggia lesion.^8^ O’Driscoll^9^, in his classification of coronoid process fractures, assigns the TFD designation as type 3.9 Treatment of TFD is mainly surgical, with open reduction and internal fixation with a plate and screws.^8,10^, but there is a lack of standardized surgical planning and execution or post-operative rehabilitation protocol^8^. Nevertheless, the result is varied from good elbow function with anatomical reduction and stability after fixation,^11^ to deficits in range of motion, progressive and disabling pain.^12-14^ The aim of this study is to evaluate the functional outcomes of surgical treatment of TFDs in a referral hospital for orthopedic trauma, with a minimum follow-up period of six months. The authors hypothesize that the standardized treatment protocol employed produces satisfactory results and is compatible with the literature.

## MATERIAL AND METHODS

A primary, longitudinal, observational study of patients with TFD surgically treated between January 2014 and November 2018 was conducted in a referral hospital for orthopedic trauma. All procedures were performed by two orthopedic surgeons with experience in elbow trauma surgery. This research was submitted to and approved by the institution's Research Ethics Committee (CAAE: 89358318.3.0000.5103). This manuscript was written according to the STROBE guideline.

### Sample

The inclusion criteria were adults with unilateral or bilateral TFD, submitted to surgical treatment with open reduction and internal fixation with plate and screws, with a post-surgical follow-up for at least six months. Patients with a history of fractures or previous trauma to the elbow, pathological fractures, and with congenital diseases in the injured limb were excluded. Those who met the selection criteria were invited for an interview, and functional and radiographic evaluation. Those who agreed to participate in the study completed a free and informed consent form and questionnaires to assess upper limb and elbow function, quality of life, and pain measurement, in addition to radiographic evaluation of the operated elbow.

### Functional outcomes

The Disability of the Arm, Shoulder and Hand (DASH) score^15^, which is a general upper limb assessment scale, and the Mayo Elbow Performance Score (MEPS)^16^, instrument for evaluation of elbow function, were used. To analyze these results in dichotomous satisfactory or unsatisfactory, the value of the minimal clinically important difference (MCID) of 10 points was used for both DASH^17^ and MEPS^18^. For quality of life (QOL), the SF-12 questionnaire^19^ was used, and following Ware's criteria^20^ for satisfactory or unsatisfactory results, we assumed a value of 50 for the physical score (PCS) and 42 for the mental score (MCS).^20^ The level of pain was verified using the visual analog scale (VAS).^21^

### Surgical technique

**Figure 1:**
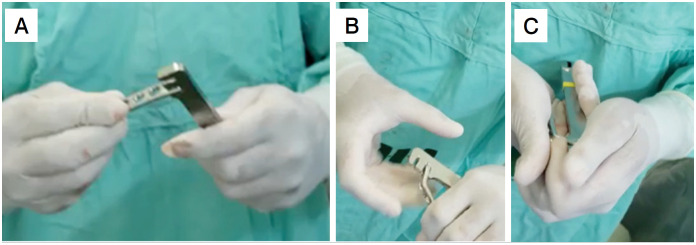
A, B, and C: Hexagon® 3.5 mm DCP modeling technique with the use of a contouring tool: A - Plate tip is placed on the widest part of the tool. B and C - Compression to bend the plate at the second hole to adapt the implant to the olecranon.

The patients were placed in a supine position with shoulder abduction of 70º and limb positioned under a radiolucent table, after regional and general anesthesia. A curved posterior incision was made around the tip of the olecranon, folding as large a fasciocutaneous flap as possible to avoid skin complications. Dissection by planes, access to the fracture with cleaning of the focus and identification of the main fragments (diaphysis, olecranon, medial and/or lateral ligament fragments) was carried out. In most cases, a 3.5mm non-locking dynamic compression plate (DCP) (Hexagon^®^ Itapira, São Paulo), contoured intra-operatively, was used for internal fixation (Figure 1 A, B, and C). In some patients, a pre-contoured locking plate from the same manufacturer was used.

**Figure 2:**
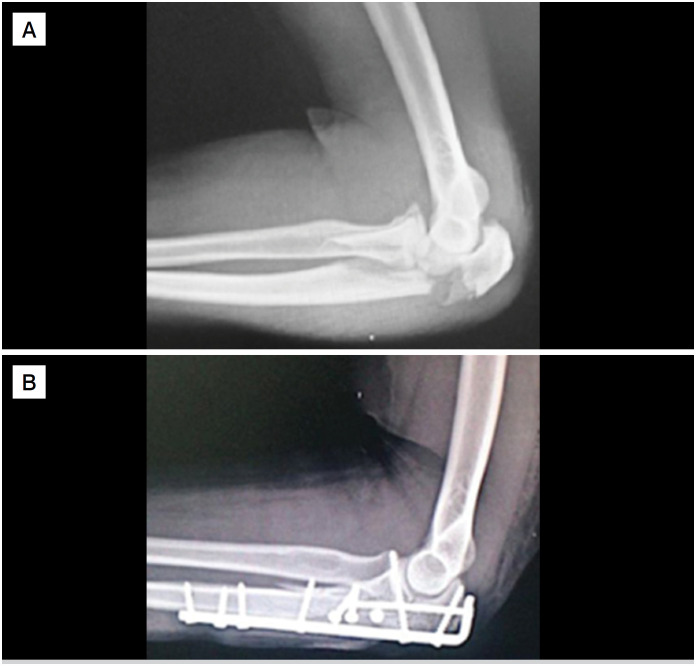
A and B: Pre and Postoperative radiograph of the profile views of the participant's left elbow showing transolecranon fracture-dislocation (A) and osteosynthesis with a 3.5 mm non locking compression plate, and lag screws (B).

Independently of the implant used, the reduction procedure began with the joint fragments and provisional fixation with intramedullary 2.0 mm Kirschner wires from the joint block to the main fragments. After that, an incision in the central portion of the triceps tendon for better accommodation of the implant, approximation of the plate using the intramedullary wire as a guide, fixation of the plate by the distal screw, followed by fixation of the proximal fragment, starting with the fragment of the coronoid process and a long 3.5 mm cortical screw in place of the temporary wire. Then, open reduction of the fragments and fixation with screws was performed or transosseous sutures with nonabsorbable wires (Figure 2) for the small fragments. Finally, assessment of the alignment of the fragments with an image intensifier, stress maneuvers in varus and valgus to assess stability, and range of motion of the elbow for joint protrusions. In the first postoperative day, active elbow movement orientation was encouraged, without load, according to pain limit, and use of a arm sling as necessary. Returns to the outpatient clinic were scheduled for the first 15- and 30-days post-operative, and then a monthly evaluation up to six months of follow-up. The physical therapy program was initiated after de 15^th^ day, according to the protocol of the institution.

### Assessment

The final assessment was carried out by an independent examiner, not involved in the surgical procedures, who proceeded to interview the participants to confirm demographic data, perform the physical examination with range of motion of the elbow and forearm, pain, DASH, MEPS and SF-12. Participants underwent imaging assessment with anteroposterior and lateral radiographs of the operated elbow, with analysis of consolidation or pseudoarthrosis, joint surface contours, presence of malunion or implant failure being carried out. This examiner also reviewed data from the participants’ medical records about the operation and its follow-up, such as time for consolidation, presence of delayed consolidation, pseudoarthrosis, infection, and failure of synthesis material.

### Statistical analysis

Quantitative variables were described using mean and standard deviation, and qualitative variables using absolute frequency and percentages. To test for differences between the groups of patients with satisfactory or unsatisfactory results according to the MEPS scale, the Student's *t*-test was used for independent samples with parametric distribution, or the Mann-Whitney U test for non-parametric samples. To test for differences in qualitative variables between groups, Fisher's exact test was used. The effect size (clinical significance) was assessed using Cohen's d (quantitative variables) or Cramer's V (qualitative variables), using the following classification for interpretation: lower Cohen's d ≤0.49; moderate 0.50 to 0.79; high ≥0.80; lower Cramer's V ≤0.29; moderate 0.30 to 0.49; high ≥0.50 (Cohen, 1992). All analyses were done using IBM SPSS version 20.0 statistical software (IBM Corp., Armonk, NY). The value of p<0.05 was adopted for statistical significance.

## RESULTS

25 individuals were included for clinical and radiographic evaluation. Most were male (60%), the mean age was 46.8 years (ranging from 21 to 89 years) (Table 1). The dominant side was affected in 40% of the cases, the mean time until surgery was eight days (ranging from 2 to 20 days), and the mean follow-up time was 25 months (ranging from 6 to 62 months). In twenty patients, a 3.5 mm Hexagon^®^ non-locking compression plate was used. Another five individuals underwent ORIF with a pre-contoured Hexagon^®^ plate. Regarding the associated procedures, in one case, an autologous bone graft (from the iliac) in the olecranon and fixation with a mini-micro fragment screw was used; seven patients had fractures of the radial head, of which one case was treated with resection of the fragment, due to its small size, in two cases ORIF of the radius was performed with 2.4mm lag screws, and in four cases arthroplasty of the radial head was performed. In one case, transosseous suture of the medial collateral ligament (MCL) was performed. Radiographic analysis showed consolidation in all cases, with no loosening, implant failure, or signs of degenerative arthritis. There was no radiographic difference between patients who used non-locking or pre-contoured implants.

**Table 1:** Comparison of characteristics of patients with transolecranon fracture-dislocation, according to the MEPS and DASH.

Factor	All (n=25)		MEPS				p-value	(ES)	DASH				p- value	ES
			Satisfactory (n= 21)		Unsatisfactory (n=4)				Satisfactory (n= 17)		Unsatisfactory			
**Age (years)**	**46.9 *±* 17.7**		**49.4 *±* 17.5**		**33.7 *±* 13.7**		**0.11**	**1.01**	**45.3 *±* 17.5**		**(n=8)**		**0.54**	**0.26**
**Sex**														
Male	15 (60.0%)		13 (86.7%)		2 (13.3%)		1.00	0.10	12 (80.0%)		3 (20.0%)		0.19	0.31
Female	10 (40.0%)		8 (80.0%)		2 (20.0%)				5 (50.0%)		5 (50.0%)			
**Affected side**														
Left	15 (60.0%)		13 (86.7%)		2 (13.3%)		1.00	0.10	9 (60.0%)		6 (40.0%)		0.40	0.21
Right	10 (40.0%)		8 (80.0%)		2 (20.0%)				8 (80.0%)		2 (20.0%)			
**Associated Injury/Fracture**														
Yes	9 (36.0%)		7 (77.8%)		2 (22.2%)		0.60	0.13	5 (55.6%)		4 (44.4%)		0.39	0.20
No	16 (64.0%)		14 (87.5%)		2 (12.5%)				12 (75.0%)		4 (25.0%)			
**Implant Type**														
Compression Plate	20 (80.0%)		16 (80.0%)		4 (20.0%)		1.00	0.22	14 (70.0%)		6 (30.0 %)		1.00	0.09
Locking Plate	5 (20.0%)		5 (100.0%)		0 (0.0%)				3 (60.0%)		2 (40.0%)			
**Comorbidities**														
Yes	8 (32.0%)		6 (75.0%)		2 (25.0%)		0.57	0.17	4 (50.0%)		4 (50.0%)		0.36	0.26
No	17 (68.0%)		15 (88.2%)		2 (11.8%)				13 (76.5%)		4 (23.5%)			
Time until surgery (days)	8.0	5.0	8.0	6.0	6.0	5.0	0.61	0.36	9.0	6.0	5.0	4.0	0.11	0.80
Follow-up time (months)	25.0	15.0	26.0	15.0	16.0	11.0	0.19	0.77	26.0	13.0	23.0	19.0	0.65	0.19

(P values calculated using Student's t test for quantitative variables and Fisher's Exact test for qualitative variables; percentages refer to the lines; ES = effect size).

**Figure 3:**
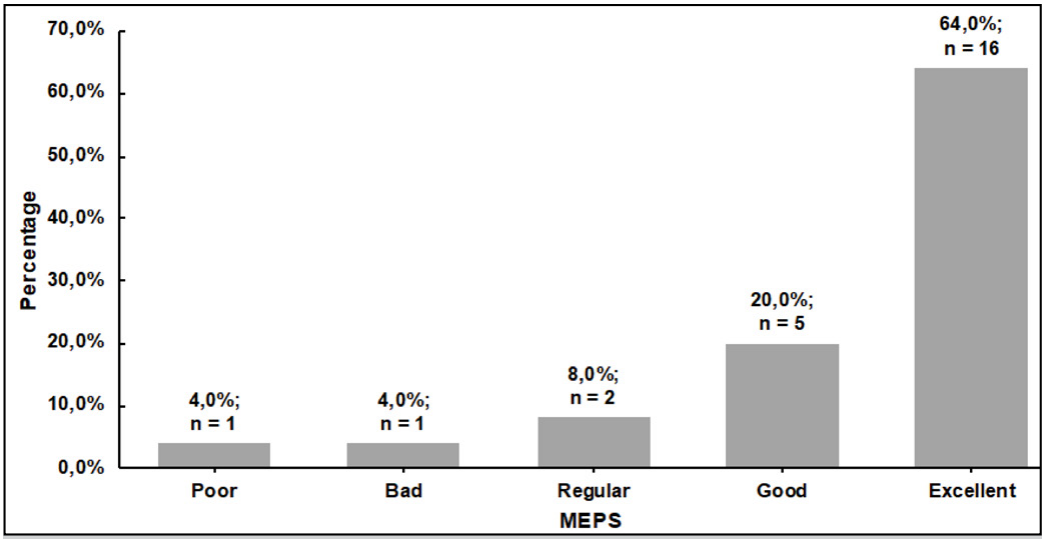
Distribution of functional results according to MEPS score.

In the functional analysis, 16 patients (68%) had excellent MEPS. (Figure 3) The MEPS results were divided into two groups: satisfactory (patients with good or excellent results) and unsatisfactory (poor, bad, and regular results). The same methodology was used for the DASH. According to this criterion, through the MEPS, 21 patients (84%) presented satisfactory results and, according to the DASH, 17 patients (68%). Table 1 shows the comparative results between patients who presented satisfactory versus unsatisfactory MEPS and DASH. The groups were similar in terms of age, sex, affected side, presence of associated fracture, presence of comorbidities, type of implant, time until surgery, and follow-up time (p>0.05). However, patients with satisfactory MEPS were younger, with trend to statistical significance (p = 0.11) and an effect size that suggests a relevant clinical difference for this variable. The TFD subgroup analysis between patients with or without radio head fractures or associated injuries demonstrated that there were no statistically significant differences between the physical exam, MEPS, or DASH variables. Table 2 presents the clinical, functional, and quality of life results of the overall sample and of the patients divided according to MEPS and DASH. Patients with satisfactory MEPS showed greater extension, flexion-extension arc, lower DASH, and greater SF-12 PCS (p<0.05). Most patients with VAS>0 were classified as unsatisfactory MEPS. Whereas the results found in relation to the DASH were not statistically significant for clinical, functional, and quality of life outcomes, except for the physical component of the SF-12.

**Table 2:** Comparison of clinical, functional, and quality of life results of patients with transolecranon fracture-dislocation, according to the MEPS and DASH.

Factor	All (n = 25)	MEPS		p	ES	DASH		p	ES
		Satisfactory (n= 21)	Unsatisfactory (n=4)			Satisfactory (n= 17)	Unsatisfactory (n=8)		
VAS									
0	21 (84.0%)	20 (95.2%)	(4.8%)	0.007*	0.70	16 (76.2%)	5 (23.8%)	0.08	0.40
≥1	4 (16.0%)	1 (25.0%)	3 (75.0%)			1 (25.0%)	3 (75.0%)		
SF-12									
MCS	45.5 ± 8.8	45.4 ± 9.1	45.6 ± 8.1	0.98	0.02	44.6 ± 8.8	47.2 ± 8.9	0.52	0.29
PCS	48.1 ± 9.7	50.4 ± 8.1	35.9 ± 8.7	0.003*	1.73	53.1 ± 5.3	37.4 ± 8.2	<0.001*	2.33
MEPS	89.6 ± 14.8	-	-	-	-	95.3 ± 8.9	77.5 ± 18.1	0.028*	1.32
DASH	16.5 ± 21.5	12.8 ± 19.5	35.8 ± 24.1	0.048*	1.06	-	-	-	-
Flexion ROM	126.4°± 15.1°	127.6°± 14.6°	120.0° ± 18.2°	0.37	0.46	129.4°± 15.3°	120.0° ± 13.4°	0.15	0.66
Extension ROM	-23.8° ± 21.9°	-17.8° ± 15.5°	-55.0° ± 26.4°	0.001*	1.78	-19.1° ± 17.1°	-33.7° ± 28.6°	0.21	0.64
Flexo-Extension ROM	102.6° ± 33.2°	109.8° ± 26.3°	65.0° ± 44.3°	0.01*	1.27	110.3° ± 27.5°	86.2° ± 40.0°	0.09	0.82
Pronation ROM	64.8° ± 14.7°	69.0° ± 8.3°	42.5° ± 22.1°	0.09	1.74	68.8° ± 9.2°	56.2° ± 20.6°	0.14	0.85
Supination ROM	68.0° ± 13.5°	67.1° ± 14.5°	72.5° ± 5.0°	0.48	0.55	70.0° ± 6.1°	63.7° ± 22.6°	0.47	0.44
Prone-Supination ROM	132.8° ± 17.7°	136.2° ± 15.3°	115.0° ± 20.8°	0.02*	1.17	138.8° ± 8.6°	120.0° ± 25.0°	0.07	1.12

(VAS: Visual Analog Pain Scale; SF12: Quality of life; p values calculated using Student's t test for quantitative variables and Fisher's Exact test for qualitative variables; ES = effect size).

## DISCUSSION

Our data analysis state that despite the complexity of the transolecranon fracture-dislocation (TFD), the average results with this standardized surgical treatment applied were satisfactory according to the functional scores. Also, in the analyzed sample, a younger age profile was observed among those with better functional results. These results are in line with the literature reports that of the surgical treatment of TFD are effective in restoring elbow congruity, but individuals generally present some functional deficit.^13,14^ Mortavizi^12^, assessing eight patients with TFD, reported satisfactory results in seven cases, according to the Morrey score. Niéto^22^, in an assessment of 11 cases of TFD, described a mean functional result of 70, according to the same score.^22^ In our sample, satisfactory results were observed in a larger number of cases (21 individuals). Mouhsine^13^, evaluating the surgical treatment of 14 patients with TFD, seven fixed with Kirschner wire and tension band, seven others with plate and screws (1/3 tubular, DCP or reconstruction), reported ten (71%) satisfactory results according to the Morrey score, and four cases of radiographic signs of degenerative arthritis in the x-ray exams.^13^ All the individuals described in our study were submitted to the same surgical protocol, and in 20 of them were used the same plate, bended with the same technique. Also, no signs of degenerative arthritis were observed, which might strengthen the standardized treatment.

Although the complex clinical presentation of TFD, no patient in our study presented clinical or radiographic instability after the applied treatment protocol. Moreover, in only one of 25 individuals a transosseous suture for medial collateral ligament was performed which raises doubts about whether TFD has its instability related to a mixed pattern of bone and ligament injury or whether the instability is mainly from the multifragmented pattern of the articular fracture, that compromises the sites of ligament insertion. According to Siebenlist^10^, the treatment of TFD should be based on the principle of stable fixation with plate and screws, given the comminuted nature of the fractures.^10^ In this sense, the standardization of bone fixation leading to stability, as demonstrated in the cases evaluated, strengthens the thesis of instability due to bone injury than soft tissue injury.

Satisfactory results were found in both the specific elbow, overall upper limb and quality of life scores. This is in line with the literature on the treatment of TFD as satisfactory, with a favorable prognosis and low incidence of complications. ^12,22^ Bailey et al^23^, analyzing the results of the surgical treatment of displaced and comminuted fractures of the olecranon in eleven patients, reported that 45% of them presented “excellent” MEPS scores.^23^ The results reported in our sample demonstrate that, according to the same evaluation, a higher percentage (64%) of excellent results were observed.

Yet, in the analysis of pain, the major percentage of the sample (84%) had a VAS of zero, coinciding with the study by Bailey et al.^23^ that found the majority of their sample without reference to pain in the evaluation. Nevertheless, mean values for the physical component of the SF-12 of 48.1 (SD 9.7) were observed, similar to that described by Bailey, who using the SF-36, found mean values of 48 (SD 12).^23^ However this author did not differentiate the quality of life measure between the functional results, unlike this sample, in which it was observed that individuals with unsatisfactory MEPS have an association with lower SF-12 PCS values, which reflects the impact of the physical alteration on an aspect of quality of life. Surgical treatment of TFD with a multiple choice of implants may lead the need for revision surgery. Ring ^8^ stated that, of his series of 17 patients, the two fixed with a 1/3 tubular plate, required revision for osteosynthesis with 3.5 mm DCP. Mortavizi^12^ reports that a case fixed with a tension band with Kirschner wire required revision for fixation with DCP.^12^ Mouhsine^13^ also reports that the use of wire fixation in the TFD required revision to fixation with plate and screws.^13^ Our results demonstrate that all TFD were treated according to the same protocol, and no need for post-operative revision was observed. Some limitations might be underscored in this study. The small sample size is related to the low incidence of TFD, it might compromise the comparison between locking and non-locking implants. The sample size, however, was similar to or greater than other studies in the literature.^8,11-13^ About the strenght aspects of our study, that the evaluation of a homogeneous sample with a low-incidence fracture, treated with the same protocol in all cases, resulted in satisfactory functional results, reinforcing the internal validity of the treatment used. Also, the low demand for osteoligamentary fixations with elbow stability in the follow-up, reinforces the theory of TFD's instability due to bone involvement, instead of soft tissues injuries.

## CONCLUSION

Surgical treatment of TFD by a standardized treatment protocol with open reduction and internal fixation with plate led to satisfactory functional results in most cases, without residual pain, and low interference in the quality of life of the patients.
